# Long-Term Effects of Suramin on Renal Function in Streptozotocin-Induced Diabetes in Rats

**DOI:** 10.3390/ijms241914671

**Published:** 2023-09-28

**Authors:** Gabriela Chyła-Danił, Kornelia Sałaga-Zaleska, Ewelina Kreft, Olaf Stumski, Aleksandra Krzesińska, Monika Sakowicz-Burkiewicz, Agnieszka Kuchta, Maciej Jankowski

**Affiliations:** 1 Department of Clinical Chemistry, Medical University of Gdańsk, Dębinki 7, 80-210 Gdańsk, Poland; gabriela_chyla@gumed.edu.pl (G.C.-D.);; 2 Department of Molecular Medicine, Medical University of Gdańsk, Dębinki 7, 80-210 Gdańsk, Poland

**Keywords:** diabetes, nephropathy, chronic kidney disease, proteinuria, streptozotocin, suramin, VEGF-A

## Abstract

In short-term diabetes (3 weeks), suramin, a drug used clinically, affects renal function and the expression of vascular endothelial growth factor A (VEGF-A), which may be involved in the pathogenesis of diabetic nephropathy, the main cause of end-stage renal disease. In the present study, we evaluated the long-term (11 weeks) effects of suramin (10 mg/kg, *i.p.*, once-weekly) in diabetic rats. Concentrations of VEGF-A, albumin, soluble adhesive molecules (sICAM-1, sVCAM-1), nucleosomes, and thrombin–antithrombin complex (TAT) were measured by ELISA, total protein was measured using a biuret reagent. Glomerular expression of VEGF-A was evaluated by Western blot, mRNA for VEGF-A receptors in the renal cortex by RT-PCR. The vasoreactivity of the interlobar arteries to acetylcholine was assessed by wire myography. Long-term diabetes led to an increased concentration of VEGF-A, TAT, and urinary excretion of total protein and albumin, and a decrease in the concentration of sVCAM-1. We have shown that suramin in diabetes reduces total urinary protein excretion and restores the relaxing properties of acetylcholine relaxation properties to non-diabetic levels. Suramin had no effect on glomerular expression VEGF-A expression and specific receptors, and on sICAM-1 and nucleosomes concentrations in diabetic rats. In conclusion, the long-term effect of suramin on the kidneys in diabetes, expressed in the reduction of proteinuria and the restoration of endothelium-dependent relaxation of the renal arteries, can be considered as potentially contributing to the reduction/slowing down of the development of diabetic nephropathy.

## 1. Introduction

Suramin—polysulfonated naphthylurea has clinical use in the treatment of parasitic diseases [1], and intensive research is being conducted into its clinical use in psychiatry in the treatment of autism [2] and in the treatment of cancer [3] and infectious diseases [4,5]. With a broad spectrum of activity, this negatively charged molecule has many extracellular and intracellular targets. It inhibits enzymes involved in glycolysis, the Krebs cycle, DNA and RNA synthesis/modification, several protein kinases and phosphatase and blocks receptors for extracellular ATP. Suramin has been shown to neutralize circulating histones that enter the bloodstream after being released from cells by apoptosis or necrosis to prevent acute endothelial dysfunction [6]. This molecule has antiviral and antibacteriophage activities and acts as a competitive inhibitor of heparin binding to a wide range of proteins. It blocks the cell surface binding of several growth factors, including the vascular endothelial growth factor (VEGF), showing remarkable inhibition of growth factors effects [7]. Vascular endothelial growth factor A (VEGF-A) is an important member of the VEGFs family of cytokines [8] that exerts a paracrine effect on endothelial cell survival, proliferation, and migration [9]. Furthermore, the autocrine VEGF-A signaling pathway is required for the maintenance of vascular homeostasis [10]. It is also required for maintaining the differentiated phenotype of endothelial cells, namely for the formation of fenestrations in glomerular endothelial cells [11]. VEGF-A also increases endothelial permeability and affects adhesion molecules, including intercellular adhesion molecule 1 (ICAM-1) and vascular cell adhesion molecule 1 (VCAM-1) [12]. The cellular response to VEGF-A is primarily controlled by the binding of VEGF-A to the cell surface receptor tyrosine kinases known as VEGFR-1 and VEGFR-2, their dimerization and transphosphorylation. VEGF-A primarily interacts with VEGFR-2 [13,14] but VEGF-A interaction with VEGFR-2 is prevented by binding to VEGFR-1 [15]. Thus, the strength of the cellular response to VEGF-A is determined by the balance between these receptors. Because VEGF-A production is stimulated by hyperglycemia, it is thought to be involved in the pathogenesis of diabetic nephropathy (DN), the major long-term complication of diabetes mellitus, which is manifested by decreased glomerular filtration rate (GFR), increased protein excretion due to increased glomerular permeability to albumin, at least in the early stages of diabetes [16]. Furthermore, at the cellular level, DN is characterized by decreased endothelial cell survival and vascular endothelial dysfunction, resulting in an imbalance between endothelium-dependent vasodilation and vasoconstriction due to decreased bioavailability of vasodilators, particularly nitric oxide (NO), or increased endothelium-derived contractile factors [17,18]. Taken together, the glomerular expression of VEGF-A and related receptors and their biological roles support the possibility of their potential involvement in DN [19]. In addition, it is likely that suramin may modify the progression of DN by altering VEGF-A binding to receptors. Consistent with this, our previous studies in short-term streptozotocin-induced diabetes showed that suramin affects renal VEGF-A with specific receptors and renal arteries’ response to acetylcholine [20,21]. In addition, suramin has been shown to reduce fibrosis and improve renal function, such as reducing proteinuria, in experimental diabetes [22,23,24]. On the other hand, adverse effects of suramin including nephrotoxicity have been reported [25]. The mechanism responsible for these effects of suramin is not fully understood, but its interference with the action of some growth factors, including VEGF, should be considered [7]. However, the experimental and clinical data obtained so far indicate that the direction of action of VEGF-A is not clearly defined depending on the duration of diabetes and the severity of DN. It has been shown that VEGF-A and VEGFR-2 are upregulated in rat kidneys during the early stages of diabetic nephropathy [26]. Both adults and children with diabetes have increased plasma VEGF levels [27]. However, the glomerular-specific depletion or overexpression of VEGF-A in mice results in glomerular pathology, suggesting the importance of a balance in VEGF-A expression [28]. Nephropathy is accelerated in diabetic animals when all VEGF-A isoforms are deleted from podocytes [29]. In addition, evidence suggests that the local interaction between VEGF-A and its receptors, but not the expression of VEGF-A, is tightly regulated during diabetes [30] and that certain levels of VEGF-A may be necessary to maintain normal glomerular function [31].

The aim of this study was to investigate the long-term effect (11 weeks) of suramin (10 mg/kg *i.p.*, weekly) on renal function, including glomerular filtration rate and urinary protein excretion, and levels of VEGF-A and its receptors and vasomotor function of renal endothelium in streptozotocin-induced diabetes.

## 2. Results

### 2.1. Experiments In Vivo

Body weight, urine output, fluid intake, and blood glucose data for rats in each experimental group are shown in Table 1. Suramin-untreated (CON) and suramin-treated (SUR) non-diabetic rats showed a significant 1.5-fold increase in body weight over 12 weeks. In diabetic rats not treated with suramin (STZ) or treated with suramin (STZ + SUR), no significant increase in body weight was observed. Suramin had no effect on glucose concentration and diuresis in non-diabetic (SUR) and diabetic (STZ + SUR) rats. Nutritional variables such as food intake, feed efficiency ratio (FER), specific rate of body-weight gain, and efficiency of food utilization by body weight (EFU_BW_) are shown in Table 2. Accordingly, diabetic rats had 2-fold higher food intake and lower specific rates of body-weight gain FER and EFU_BW_ compared to non-diabetic rats. In both diabetic and non-diabetic rats, suramin had no significant effect on these nutritional variables.

### 2.2. Experiment In Vitro

**Figure 1:**
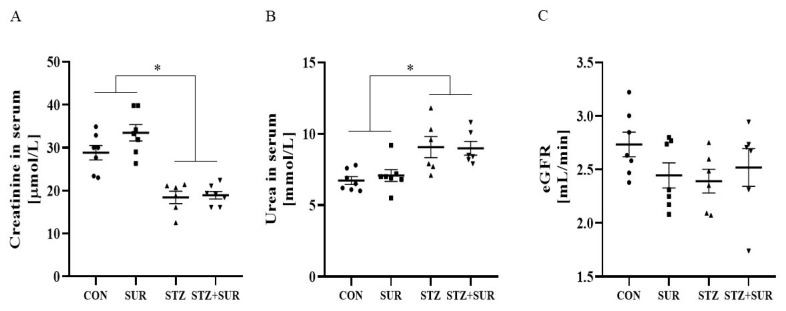
Effects of suramin on serum creatinine (**A**) and urea (**B**) concentrations and the estimated glomerular filtration rate (eGFR) (**C**) in non-diabetic and diabetic rats. Non-diabetic rats (CON) and streptozotocin-induced diabetic rats (STZ) were injected with suramin (10 mg/kg, *i.p.*, SUR) or saline weekly for 11 weeks, starting one week after streptozotocin or citrate buffer injection (week 0). Week 12 results are presented as individual data points with mean ± standard error of the mean. Statistical significance: * *p* < 0.05 vs. indicated group (two-way ANOVA).

To test the potential long-term effect of suramin in diabetes on glomerular filtration, we estimated the glomerular filtration rate based on serum creatinine and urea concentrations and body weight. Serum creatinine (A) and urea (B) concentrations and the estimated glomerular filtration rate (C) are shown in Figure 1. Two-way ANOVA showed no effect of suramin on the above parameters (serum creatinine *p* = 0.1965, serum urea *p* = 0.6550, and estimated glomerular filtration rate *p* = 0.1273). The concentration of creatinine was decreased by 40% in diabetic versus control rats (18.71 ± 0.78 µmol/L vs. 31.16 ± 1.39 µmol/L, *p* < 0.0001) (Figure 1A), whereas the concentration of urea was increased by 24% in diabetic versus control rats (9.03 ± 0.42 mmol/L vs. 6.90 ± 0.24 mmol/L, *p* = 0.0003) (Figure 1B). In addition, as shown in Figure 1C, the estimated glomerular filtration rate was similar in all experimental groups.

**Figure 2:**
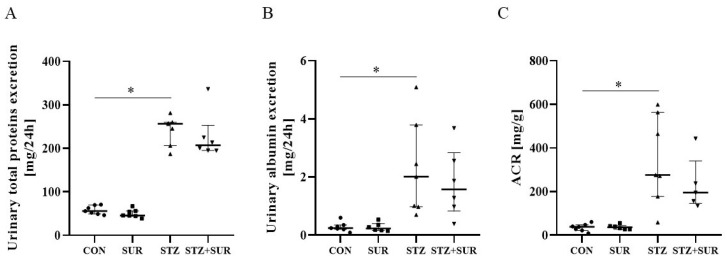
Effects of suramin on urinary excretion of total protein (**A**) and albumin (**B**) and urinary albumin–creatinine ratio, ACR (**C**) in non-diabetic and diabetic rats. Non-diabetic rats (CON) and streptozotocin-induced diabetic rats (STZ) were injected with suramin (10 mg/kg, *i.p.*, SUR) or saline (non-SUR) weekly for 11 weeks, starting one week after streptozotocin or citrate buffer injection (week 0). Week 12 results are presented as individual data points with median and 25th–75th percentiles. Statistical significance: * *p* < 0.05 vs. indicated group (Dunn’s multiple comparison tests).

Figure 2 shows the results of urinary excretion of total protein (Figure 2A), albumin (Figure 2B), and albumin/creatinine ratio (ACR) (Figure 2C). There was no significant difference in urinary excretion of total protein (Figure 2A), albumin concentration (Figure 2B), and albumin/creatinine ratio (Figure 2C) between non-suramin and suramin groups in both non-diabetic and diabetic rats. Long-term diabetes is characterized by an approximately 5-fold increase in total protein excretion (256 <206; 260> mg/24 h in diabetic rats and 56 <49; 70> mg/24 h in non-diabetic rats, *p* = 0.0164), an 8-fold increase in albumin excretion (2.01 <0.97; 3. 83> mg/24 h in diabetic rats and 0.25 <0.20; 0.35> mg/24 h in non-diabetic rats, *p* = 0.0088), and a 7-fold albumin:creatinine ratio (276 <179; 563> mg/g in diabetic rats and 38 <23; 48> mg/g in non-diabetic rats, *p* = 0.0038).

**Figure 3:**
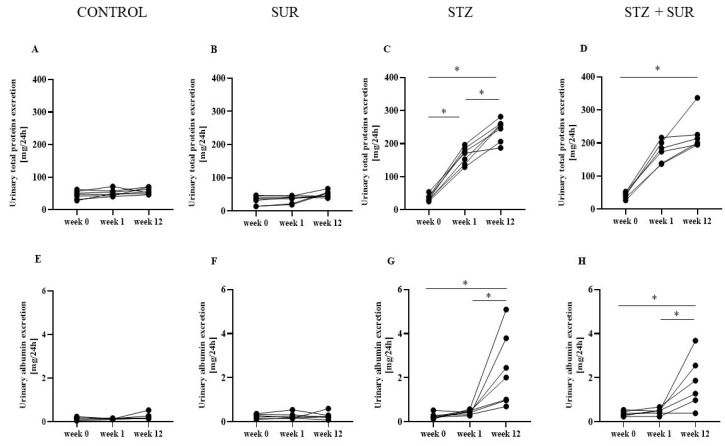
Effect of suramin on urinary excretion of protein in non-diabetic and diabetic rats. Non-diabetic rats and streptozotocin-induced diabetic rats were injected with suramin (10 mg/kg, *i.p.*, SUR) or saline weekly for 11 weeks, starting 1 week after streptozotocin or citrate buffer injection (week 0). (**A**,**E**)—Non-diabetic (CONTROL); (**B**,**F**)—Non-diabetic rats treated with suramin (SUR); (**C**,**G**)—Diabetic rats (STZ); (**D**,**H**)—Diabetic rats treated with suramin (STZ + SUR). Results are presented as single data points obtained at weeks 0, 1, and 12. Statistical analysis was performed using a two-way ANOVA. Significance: * *p* < 0.05 vs. indicated week (Tukey’s post hoc test).

Data from a detailed analysis of total protein and albumin excretion at three time points of the experiments are presented in Figure 3. There were no significant changes in urinary albumin and total protein excretion in non-diabetic rats, either in the control group (Figure 3A,E) or in the suramin-treated group (Figure 3B,F). During the first week of the experiment, diabetic rats significantly increased total protein excretion (Figure 3C,D) but not albumin excretion (Figure 3G,H). The following 11 weeks of diabetes (week 12) resulted in a further significant increase in total urinary protein excretion only in the suramin-naive group (Figure 3C,D). Urinary albumin excretion was significantly higher after 12 weeks of diabetes in both suramin-treated and untreated rats (Figure 3G,H).

**Figure 4:**
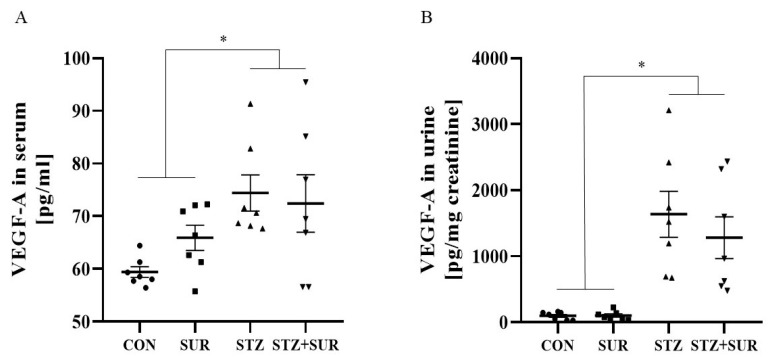
Effects of suramin serum VEGF-A concentration (**A**) and urinary VEGF-A excretion (**B**) in non-diabetic and diabetic rats. Non-diabetic rats (CON) and streptozotocin-induced diabetic rats (STZ) were injected with suramin (10 mg/kg, *i.p.*, SUR) or saline weekly for 11 weeks, starting one week after streptozotocin or citrate buffer injection (week 0). Week 12 results are presented as individual data points with mean ± standard error of the mean. Statistical significance: * *p* < 0.05 vs. indicated group (two-way ANOVA).

Next, the serum VEGF-A concentration and urinary VEGF-A excretion were then measured, and the results are shown in Figure 4. Suramin treatment did not affect the levels of the above parameters and had no effect on the detected differences between non-diabetic and diabetic rats (serum VEGF-A concentration *p* = 0.2344 and urinary VEGF-A excretion *p* = 0.4534). The serum concentration of VEGF-A in diabetic rats was significantly increased by 15% compared to the control group (73.44 ± 3.11 pg/mL vs. 62.67 ± 1.54 pg/mL, *p* = 0.005, Figure 4A). According to the data presented in Figure 4B, the urinary excretion of VEGF-A was approximately 15-fold higher in diabetic rats compared to control rats (1459.0 ± 232.0 pg/mg creatinine vs. 100.3 ± 15.8 pg/mg creatinine, *p* < 0.0001).

**Figure 5:**
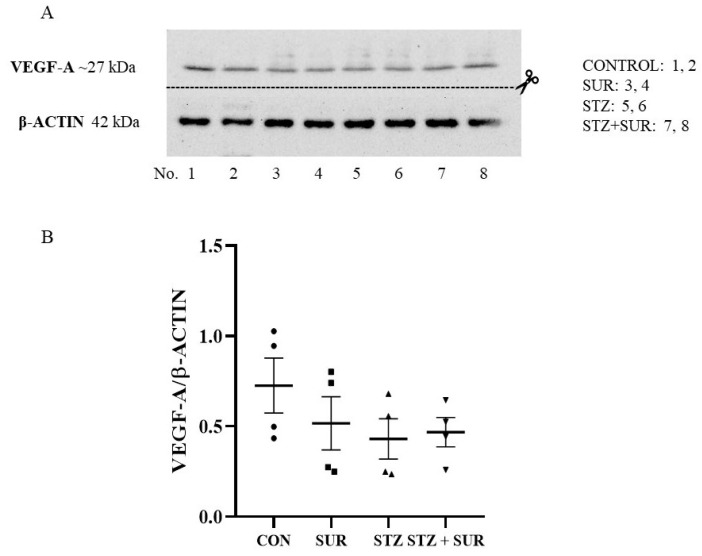
Effects of suramin on the expression of VEGF-A protein in glomeruli isolated from non-diabetic and diabetic rats. Non-diabetic rats (CON) and streptozotocin-induced diabetic rats (STZ) were injected with suramin (10 mg/kg, *i.p.*, SUR) or saline weekly for 11 weeks, starting one week after streptozotocin or citrate buffer injection (week 0). Results at week 12 are shown. (**A**) Example of immunoblot showing VEGF-A protein expression in isolated glomeruli; lanes 1, 2: non-diabetic Suramin untreated (CONTROL); lanes 3, 4: non-diabetic Suramin treated (SUR); lanes 5, 6: diabetic Suramin untreated (STZ); lanes 7, 8: diabetic Suramin treated (STZ + SUR). (**B**) Densitometry scan: relative optical density expressed as VEGF-A/β-actin ratio. VEGF-A/β-actin expression results are presented as individual data points with mean ± standard error of the mean. Statistical analysis was performed using a two-way ANOVA. CON *n* = 4; SUR *n* = 4; STZ *n* = 4; STZ + SUR *n* = 4.

Figure 5 shows data on VEGF-A protein levels in isolated glomeruli. There was no difference in VEGF-A/β-actin levels between diabetic and control glomeruli (0.45 ± 0.06 vs. 0.62 ± 0.11, *p* = 0.1992), and suramin did not affect the expression of VEGF-A/β-actin levels (*p* = 0.3462).

**Figure 6:**
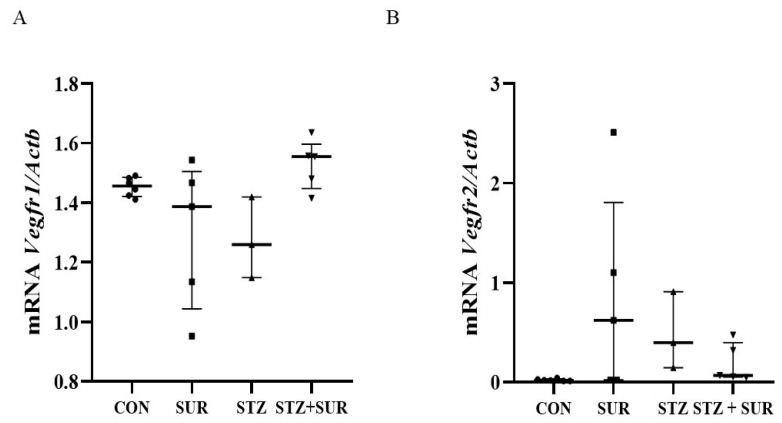
Effects of suramin on *Vegfr1* (**A**) and *Vegfr2* (**B**) mRNA expression in the renal cortex of non-diabetic and diabetic rats. Non-diabetic rats (CON) and streptozotocin-induced diabetic rats (STZ) were injected with suramin (10 mg/kg, *i.p.*, SUR) or saline weekly for 11 weeks, starting one week after streptozotocin or citrate buffer injection (week 0). Week 12 results are presented as individual data points with median and 25th–75th percentiles. The β-actin gene (*Actb*) reference transcript was used for quantification of mRNA expression. Statistical analysis was performed using Dunn’s multiple comparison test. CON *n* = 6; SUR *n* = 5; STZ *n* = 3; STZ + SUR *n* = 5.

The expression of VEGF-A mRNA receptors (*Vegfr1* and *Vegfr2*) in the renal cortex was then quantified by RT-PCR (Figure 6). According to the data presented, neither diabetes (*Vegfr1* 1.46 <1.42; 1.49> vs. 1.26 <1.15; 1.42>, *p* = 0.7895; *Vegfr2* 0.017 <0.011; 0.031> vs. 0.395 <0.143; 0.908>, *p* = 0.0637) nor suramin (*Vegfr1* 1.46 <1.42; 1.49> vs. 1.39 <1.04; 1.50>, *p* > 0.9999; *Vegfr2* 0.017 <0.011; 0.031> vs. 0.622 <0.022; 1.806>, *p* = 0.0677) had any effect on mRNA receptor levels (Figure 6A,B).

**Figure 7:**
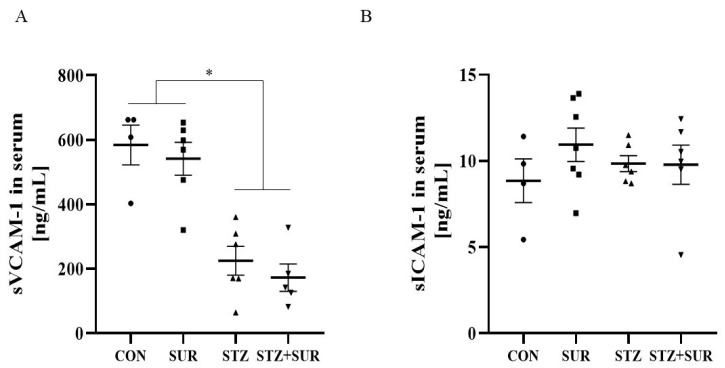
Effects of suramin on serum concentrations of soluble vascular cell adhesion molecule-1, sVCAM-1 (**A**) and soluble intercellular adhesion molecule-1, sICAM-1 (**B**) in non-diabetic and diabetic rats. Non-diabetic rats (CON) and streptozotocin-induced diabetic rats (STZ) were injected with suramin (SUR) or saline weekly for 11 weeks, starting one week after streptozotocin or citrate buffer injection (week 0). Results at week 12 are presented as individual data points with mean ± standard error of the mean. The results are presented as individual data points with mean ± standard error of the mean. Statistical significance: * *p* < 0.05 vs. indicated group (two-way ANOVA).

Further experiments were performed to measure the serum soluble forms of adhesion molecules (VCAM-1, ICAM-1), as VEGF-A may influence the expression of these molecules (sVCAM-1, sICAM-1), and the results are shown in Figure 7. There was a 48% reduction in sVCAM-1 concentration in diabetic rats compared to non-diabetic rats (525.9 ± 83.8 ng/mL vs. 1010.0 ± 64.9 ng/mL, *p* < 0.0001) (Figure 7A) and suramin treatment did not affect sVCAM-1 concentration and showed differences between non-diabetic and diabetic rats (Figure 7A) (*p* = 0.9242). Neither diabetes (9.81 ± 0.58 ng/mL vs. 10.18 ± 0.80 ng/mL, *p* = 0.9361) nor suramin (10.40 ± 0.72 ng/mL vs. 9.44 ± 0.55 ng/mL, *p* = 0.3205) treatment affected serum sICAM-1 concentrations and there were no significant differences in its concentrations between the study groups (Figure 7B).

**Figure 8:**
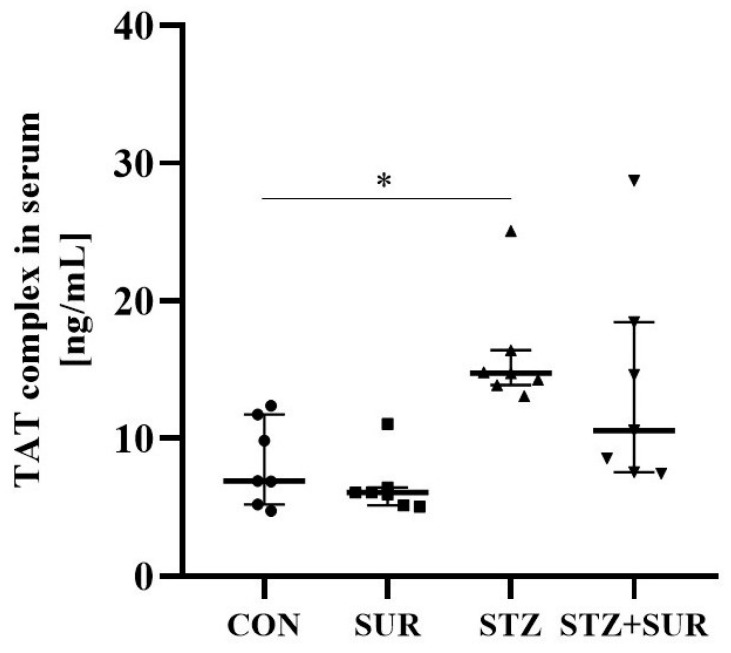
Effect of suramin on serum concentration of thrombin–antithrombin complex (TAT) in non-diabetic and diabetic rats. Non-diabetic rats (CON) and streptozotocin-induced diabetic rats (STZ) were injected with suramin (10 mg/kg, *i.p.*, SUR) or saline (non-SUR) weekly for 11 weeks, starting one week after streptozotocin or citrate buffer injection (week 0). Week 12 results are presented as individual data points with median and 25th–75th percentiles. Statistical significance: * *p* < 0.05 vs. indicated group (Dunn’s multiple comparison test).

Figure 8 shows the median thrombin–antithrombin complex (TAT) concentration between the 25th and 75th percentiles. Long-term diabetes was characterized by an approximately 2-fold increase in TAT concentration compared to non-diabetes in both non-suramin (14.76 <13.87; 16.40> ng/mL vs. 6.91 <5.21; 11.75> ng/mL, *p* = 0.031) and suramin (10.58 <7.55; 18.47> ng/mL vs. 6.08 <5.14; 6.46> ng/mL, *p* = 0.056) rats. No effect of suramin was observed in non-diabetic rats (6.91 <5.21; 11.75> ng/mL in non-suramin rats vs. 6.08 <5.14; 6.46> ng/mL in suramin rats, p > 0.999) or diabetic rats (14.76 <13.87; 16.40> ng/mL vs. 10.58 <7.55; 18.47> ng/mL, *p* > 0.999).

**Figure 9:**
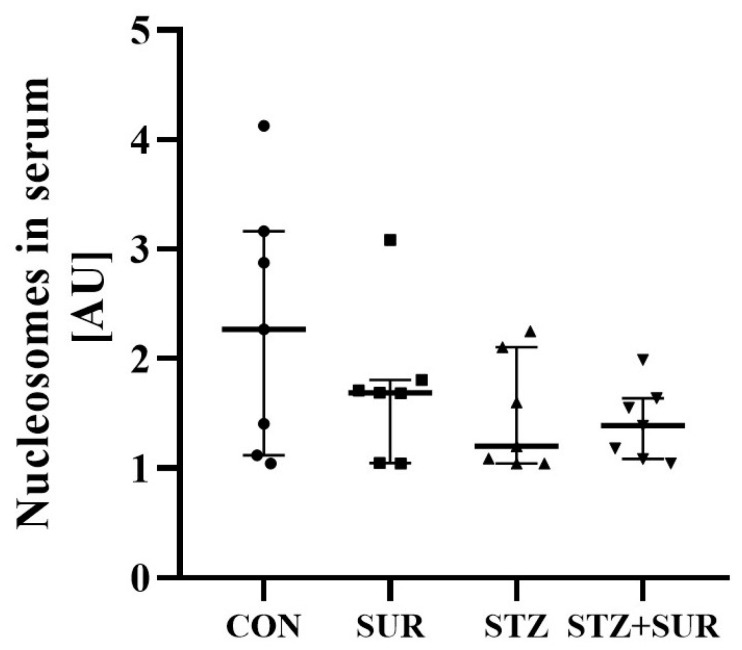
Effect of suramin on serum concentration of nucleosomes in non-diabetic and diabetic rats. Non-diabetic rats (CON) and streptozotocin-induced diabetic rats (STZ) were injected with suramin (10 mg/kg, *i.p.*, SUR) or saline (non-SUR) weekly for 11 weeks, starting one week after streptozotocin or citrate buffer injection (week 0). Week 12 results are presented as individual data points with median and 25th–75th percentiles. Statistical analysis was performed using Dunn’s multiple comparison test.

Serum nucleosome concentrations are shown in Figure 9 and, according to the data presented, neither diabetes (2.27 <1.12; 3.16> AU vs. 1.20 <1.05; 2.11> AU, *p* = 0.910) nor suramin (2.27 <1.12; 3.16> AU vs. 1.69 <1.05; 1.81> AU, *p* > 0.999) had any effect on this parameter.

### 2.3. Experiments Ex Vivo

**Figure 10:**
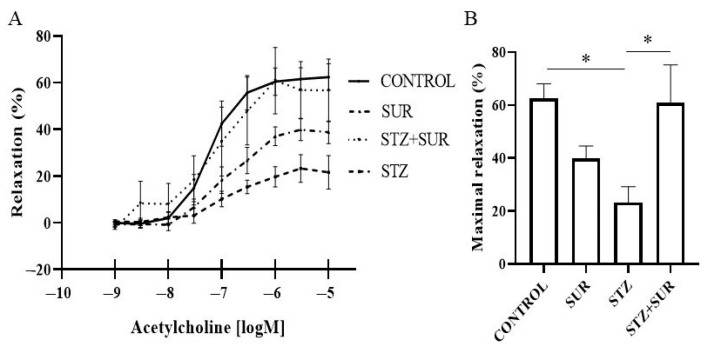
Relaxant effect of acetylcholine on the interlobar artery isolated from non-diabetic and diabetic rats treated with suramin. Concentration-dependent effect of acetylcholine on phenylephrine-precontracted interlobar artery (**A**) and maximal effect of acetylcholine (**B**). Non-diabetic rats (CONTROL) and streptozotocin-induced diabetic rats (STZ) were injected with suramin (SUR) or saline weekly for 11 weeks, starting 1 week after streptozotocin or citrate buffer injection (week 0). Results at week 12 are presented as mean ± standard error of the mean. Statistical significance: B. * *p* < 0.05 vs. indicated group, two-way ANOVA with Tukey’s post hoc test.

In the next step, the effect of suramin on the relaxation capacity of the arteries was investigated by wire myography of renal interlobar arteries (ILA) by studying the effect of acetylcholine (10^−9^–10^−5^ M) on phenylephrine-precontracted ILA isolated from non-diabetic and diabetic rats treated or untreated with suramin (Figure 10). The concentration-dependent effects of acetylcholine were noted for each experimental group (Figure 10A), and maximum relaxation values are shown in Figure 10B. In the control group (non-diabetic untreated with suramin), a minimal relaxing effect of approximately 42% (*p* < 0.0001) was observed with 10^−7^ M acetylcholine, and a maximal relaxing effect of approximately 62% (*p* < 0.001) with 10^−5^ M acetylcholine. Administration of suramin to non-diabetic rats shifted the minimum effective concentration to 10^−6^ M with an effect of approximately 37% (*p* = 0.0119), and a maximum effect of approximately 39% (*p* = 0.0197) was observed at 10^−5.5^ M. In diabetic rats, the minimum effective concentration of acetylcholine was 10^−6^ M, which induced a relaxation of approximately 19% (*p* = 0.0109), but a maximum effect of approximately 23% (*p* = 0.0042) was observed at 10^−5.5^ M, which was significantly reduced compared to control rats (*p* = 0.0110, Figure 10B). However, administration of suramin to diabetic rats resulted in a maximal relaxation of acetylcholine of approximately 61% (*p* = 0.0105) at 10^−6^ M at a minimum effective concentration of 10^−6.5^ M, and this value was not significantly different from the value of maximal relaxation observed in control rats. Administration of suramin to diabetic animals restored the relaxing properties of acetylcholine to those found in non-diabetic animals.

## 3. Discussion

We have shown that long-term streptozotocin-induced diabetes in rats is associated with (a) increased urinary excretion of VEGF-A, total protein and albumin, (b) increased concentrations of VEGF-A and the thrombin–antithrombin complex in the blood, (c) decreased blood concentration of soluble adhesion molecule vascular cells 1 (sVCAM-1), (d) attenuation of endothelium-dependent vasorelaxation. Importantly, we have shown that prolonged administration of suramin to diabetic rats (12 weeks) leads to a decrease in total urinary protein excretion and restoration of endothelium-dependent vasodilatation to values observed in non-diabetic animals. However, we did not observe significant changes in the expression of VEGF-A in the glomeruli, *Vegfr1* and *Vegfr2* mRNA in the renal cortex, the concentration of soluble intercellular adhesion molecule 1 (sICAM-1), and nucleosomes.

The dose of suramin in the treatment of the hemolymphatic stage of sleeping sickness is in the range of 4–20 mg/kg administered weekly; hence, the dose of suramin used in our experiments (10 mg/kg) is similar to that used in humans [4]. Suramin accumulates mainly in the kidneys and has a long half-life (44–54 days), requiring weekly administration [32]. A higher dose of suramin (i.e., 1 g) may lead to renal complications manifested as proteinuria [25]. In our experiments, suramin administered to control animals for 12 weeks on a weekly basis did not affect total urinary protein and albumin excretion. In addition, suramin had no effect on the glomerular filtration rate estimated from serum creatinine and urea. Taken together, these results indicate that suramin at the dose used does not adversely affect renal function. Since the progression of diabetic nephropathy is correlated with the amount of protein excreted in the urine, drugs that reduce urinary protein excretion may have a nephroprotective effect [33]. 

The progression of diabetic nephropathy is correlated with the amount of protein excreted in the urine. We have observed that diabetes is associated with increased urinary excretion of total protein and albumin. Increased urinary albumin excretion suggests glomerular damage due to increased glomerular filter permeability, which in turn is most likely due to the loss of podocytes. However, total urine proteins, the main fraction of which are proteins with a molecular weight of 18–19 kDa called major urine proteins (MUPs), are synthesized in the liver, bind pheromones in the blood and about 60% of MUPs pass through the glomerular filter and are reabsorbed in the proximal tubule [34,35]. Administration of suramin to diabetic rats for 11 weeks resulted in decreased MUPs, but not albumin. These results suggest that suramin may affect renal protein turnover at the tubular level, but probably not at the glomerular one. The answer to the question about the mechanism of action of suramin and changes in the amount of MUPs requires further research. Nevertheless, it can be assumed that reducing the amount of protein excreted in the urine may protect the kidneys and delay the development of diabetic nephropathy. Our current results on the effect of suramin in diabetic animals are in line with previous findings, as suramin administered intraperitoneally at 10 mg/kg once a week for two weeks has been shown to prevent proteinuria, attenuate renal fibrosis, and glomerular damage in a residual kidney model of chronic kidney disease [22]. 

We have demonstrated a beneficial effect of suramin on the endothelium-dependent vasomotor function of small renal arteries. Endothelial cells produce and secrete vasoactive factors, such as NO, and prostacyclin, which affect the tone of vascular smooth muscle and thus regulate blood flow. Reduced NO function is a hallmark of microcirculatory disease and appears to be a dominant factor in endothelium-dependent vasodilation dysfunction in diabetes [36,37]. We found, in agreement with other investigators, that the renal endothelium-dependent relaxation response to acetylcholine was attenuated in long-term diabetes to one-third the level of response seen in non-diabetic rats [38,39]. The diabetes-induced mechanism of acetylcholine impairment involves reduced NO bioavailability and disconnection of endothelial nitric oxide synthase, leading to the formation of superoxide anions instead of NO [40]. The potential antioxidant effect of suramin was demonstrated to protect collagen-induced arthritis [41]. In the present study, we demonstrated that prolonged administration of suramin restored endothelium-mediated relaxation of renal interlobular artery vasodilation in diabetes. The effect of suramin probably does not depend on the duration of diabetes, as we have previously shown a similar effect in short-term diabetes [21]. The mechanism of suramin action is not fully understood, but the VEGF-A/VEGFR axis may be involved because chronic VEGF-A treatment preserves acetylcholine-induced vascular responses in streptozotocin-induced diabetic rats, and this effect is accompanied by normalization of superoxide anion levels and NO and the expression of endothelial NO synthase [42]. On the other hand, hyperglycemia-dependent endothelium dysfunction is associated with enhanced thrombin formation and platelet activation [43]. Once generated, thrombin is inhibited upon binding to antithrombin, thus forming a stable thrombin–antithrombin (TAT) complex. Thus, TAT complexes are considered a marker of in vivo intravascular thrombin generation [44]. In our experiments, we observed elevated levels of TAT in the blood of rats with diabetes and no effect of suramin on this parameter in non-diabetic and diabetic rats. This suggests that effects on endothelial cells may affect NO signaling but are not involved in thrombin formation pathways. 

It is postulated that VEGF-A is involved in the pathogenesis of diabetic nephropathy (DN); however, its actual role is unclear, indicating its adverse effect on the permeability of the glomerular filter for albumin and a beneficial effect on others [45]. With an incomplete understanding of late events in the pathogenesis of DN, we decided to use the long-term streptozotocin-induced diabetes model to investigate the role of VEGF-A in the late stages of the disease. In addition, we used suramin as a pharmacological tool to potentially modify VEGF-A expression or concentration, as we have previously shown that suramin in short-term diabetes increases urinary excretion of VEGF-A without altering its blood concentration [20]. We measured the concentration of VEGF-A in the blood and its excretion in the urine, and both parameters were elevated. Increased concentration of this cytokine in the blood was previously observed in chronic experimental diabetes. In addition, a systematic review and meta-analysis showed that hyperglycemia in diabetes is strongly associated with elevated VEGF-A [27]. Since urinary and systemic VEGF-A correlate neither with glomerular VEGF expression nor with the severity of glomerular lesions [46] we assessed VEGF-A protein levels in the glomeruli of long-term diabetic rats. In glomeruli isolated from 12-week-old diabetic rats, we did not observe any significant changes in VEGF-A protein levels. In addition, no significant effect of suramin was observed on VEGF-A protein levels in glomeruli isolated from rats without or with diabetes. The potential biological effects of VEGF-A should be regarded based on VEGF-A protein expression and specific receptor levels and duration of diabetes [30]. Therefore, we measured renal cortex mRNA expression for two receptors: *Vegfr1* and *Vegfr2*. We did not note any significant changes in mRNA expression of both receptors in diabetes or under the influence of suramin. The effect of long-term diabetes on *Vegfr2* has been described previously [26]. 

In physiological equilibrium, endothelial cells are quiescent, expressing low levels of adhesion molecules (ICAM-1 and VCAM-1) [47]. Furthermore, their dysfunction is characterized by elevated levels of these molecules, resulting in the adhesion of leukocytes to endothelial cells during atherosclerosis, diabetes, and hypertension [48,49]. The release of ectodomain adhesion molecules from the endothelial cell surface results in the formation of soluble forms, sICAM-1 and sVCAM-1, and the rate of this process is related to the increased expression of membrane proteins [50]. VEGF-A stimulates the expression of ICAM-1 and VCAM-1 [12]. In the current study, we did not notice significant changes in sICAM-1 and VCAM-1 in the blood of rats with diabetes. These results suggest that systemic endothelial inflammation is unlikely in long-term streptozotocin-induced diabetes [51]. Furthermore, in our experiments, suramin did not affect the concentration of sICAM-1 or sVCAM-1 in non-diabetic and diabetic rats, but it should be observed that increased expression of ICAM-1 protein in the diabetic renal cortex has previously been shown and suramin completely blocked the increased expression of ICAM-1. However, such measurements were not made in this study [23]. Assessment of ICAM-1 and VCAM-2 expression in the kidneys and maturement of sICAM-1 and sVCAM-1 may contribute to expanding knowledge about the involvement of these cytokines in the pathogenesis of diabetic nephropathy and potential action of suramin, especially after it was found that sICAM-1 and ICAM-1 in urine are not useful for assessing ICAM-1 expression and inflammatory activity in the kidneys [52].

We are aware of the limitations of our study, which may affect the results and their interpretation. The first limitation of our study is the use of an animal research model, which is not a complete representation of the pathogenesis of diabetes in humans. It should be noted that the results obtained in the animal model may differ from the results obtained in later stages of clinical trials. However, the use of rats as an experimental model to study the mechanisms of damage to the endothelial function of the renal arteries and the function of the filtration barrier is determined by the fact of high anatomical similarity and the course of physiological processes in the rat and human kidneys. As a result, rats have become the classic animal model for biochemical and physiological kidney experiments. Study limitations include the fact that our study is based on a streptozotocin-induced type 1 diabetes model. This is not a model based on autoimmune pathogenesis, but on selectively damaged beta islets of the pancreas by means of a pharmaceutical. However, this model of type 1 diabetes is used in many research studies and is well described.

In conclusion, prolonged administration of suramin has beneficial effects on urinary total protein excretion and endothelium-dependent relaxation of the renal arteries in diabetic rats, suggesting that suramin may have potential properties limiting the development of renal dysfunction in diabetes.

## 4. Materials and Methods

### 4.1. Animals

The animal experiment protocol was approved by the Local Ethical Committee for animal experiments in Bydgoszcz (Consent No. 44/2019, 12 December 2019). The experiments were performed in male Wistar rats (Tri-City Academic Laboratory Animal Center, Gdansk, Poland), weighing 200–250 g, 8–10 weeks old at the start of the experiments. They were maintained on a 12 h light/12 h dark cycle and fed a standard granulated diet (Labofeed B, Kcynia, Poland) and water ad libitum. Wistar rats were given a single intraperitoneal dose of streptozotocin (60 mg/kg, *i.p.*) or citrate buffer. The rats were randomly assigned to one of four groups (*n* = 7 rats per group):

1. Non-diabetic group (CON); citrate buffer was injected on the first day of the experiment (week 0), followed by weekly saline injections for 11 weeks.
2. Suramin-treated non-diabetic group (SUR); suramin (10 mg/kg, *i.p.*) was injected weekly for 11 weeks, starting 1 week after citrate buffer administration.
3. Diabetes group (STZ); streptozotocin was injected on the first day of the experiment (week 0), followed by weekly saline injections for 11 weeks.
4. Suramin-treated diabetes group (STZ + SUR); suramin (10 mg/kg, *i.p.*) was injected weekly for 11 weeks, starting one week after streptozotocin administration.

**Figure 11:**
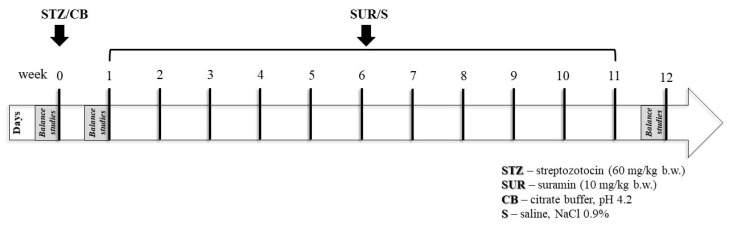
Schema of experimental protocol.

Streptozotocin and Suramin were injected in a 500 µL volume. The experiments were performed on rats with a blood glucose concentration in the tail of more than 11 mM, measured 7 days after the injection of STZ. Three 24 h metabolic cage urine samples (Tecniplast, Villa Carcina, Italy) were collected: one day before STZ injection (week 0), one day before the first SUR injection (week 1), and one day before the end of the study (week 12). Urine was collected into tubes containing protease inhibitors (10^−6^ M leupeptin, 5 × 10^−4^ M PMSF) and 3 × 10^−3^ M NaN_3_. At the end of the experiment on day 77, all animals received an overdose of anesthetic, the heart was punctured, and blood was collected for serum. The experimental protocol is shown in Figure 11.

### 4.2. Renal Interlobar Artery Preparation

Animals, anesthetized with inhalation of isoflurane (2.5%, flow rate 0.5 L/min), were killed with an intraperitoneal injection of a lethal dose of pentobarbital (120 mg/kg body weight). Kidneys were immediately removed and placed in cold preparation solution (in mM): 146 NaCl, 5.5 glucose, 5 HEPES, 4.5 KCl, 1.2 NaH_2_PO_4_, 1.0 MgSO_4_, 0.1 CaCl_2_, 0.025 EDTA-Na, and pH 7.4. The kidney was dissected along the major axis of the tubular and vascular structures. Arterial sections (approximately 2 mm in length) were isolated from the interlobar arteries.

### 4.3. Vascular Response Measurements

The interlobar artery (ILA) was mounted on a 40 μm diameter stainless steel wire in a multi-chamber small vessel wire myograph (model 620M, DMT, Hinnerup, Denmark) filled with cold preparation solution. The buffered preparations were heated to 37 °C and ventilated with carbogen (95% O_2_, 5% CO_2_) for 20 min prior to testing. After assembly, the solution was changed to the experimental solution (in mM): 119 NaCl, 25 NaHCO_3_, 5.5 glucose, 4.7 KCl, 2.5 CaCl_2_, 1.2 KH_2_PO_4_, 1.2 MgSO_4_, 0.03 EDTA-Na, and pH 7.4. Under an unrestricted flow of carbogen, the myograph was heated to 37 °C. The vessels were then adjusted to a distensibility equivalent to 90% of the distensibility at 100 mmHg intramural pressure. The viability of the ILA was demonstrated by applying a high potassium solution (in mM): 123.7 KCl, 25 NaHCO_3_, 5.5 glucose, 2.5 CaCl_2_, 1.2 KH_2_PO_4_, 1.2 MgSO_4_, 0.03 EDTA-Na, and pH 7.4. Endothelium-dependent vasorelaxation was assessed by cumulative concentration-response relationships to acetylcholine (ACh) (10^−9^–10^−5^ M) in arteries precontracted with phenylephrine to 50–70% of maximal arterial force in a potassium-rich solution. Arterial relaxation was induced on stable phenylephrine-induced precontracted ILA. Data were digitized using the LabChart 8 system (ADinstruments, Dunedin, New Zealand).

### 4.4. Relative Quantitative Real-Time RT-PCR Assay 

The isolation of RNA from the kidney cortex was performed by the Chomczynski method [53]. In brief, the kidney tissue was homogenized in a sterile tube with 1 mL of RNA extraction buffer (TRIzol Reagent, Gibco, Grand Island, NY, USA). The extraction was started by adding 250 μL chloroform. After thorough agitation, each sample was incubated at 4 °C for 15 min and centrifuged (10,000× *g*) at 4 °C for 15 min. The supernatant was transferred to a new tube and isopropanol was added in a 1:2 ratio (isopropanol: RNA Extracol, *v*/*v*). The RNA was precipitated overnight at −20 °C and then centrifuged (10,000× *g* at 4 °C for 15 min). The RNA pellet was first washed with 99.8% ethanol and then with 75% ethanol (*v*/*v*), air-dried and dissolved in nuclease-free water (15–20 μL), and stored at −20 °C. The amount of isolated RNA has been set by fluorometry using the Qubit RNA HS assay kit by Qubit^®^ 2.0 Fluorometer (Life Technologies, Grand Island, NY, USA) according to the manufacturer’s instructions. The expression levels of the *Vegfr1* and *Vegfr2* genes were determined by real-time RT-PCR, which was performed on a Light Cycler 480 II (Roche Diagnostic GmbH, Mannheim, Germany) using the Path-ID Multiplex One-Step RT-PCR Kit (Thermo Fisher Scientific, Waltham, MA, USA) and the corresponding Universal Probe Library for rats (Roche Applied Science, Penzberg, Germany). Primer and probe sequences used for *Vegfr1* and *Vegfr2* are listed in Table 3. The reaction mixture contained 5 μL Multiplex RT-PCR Buffer, 1 μL Multiplex Enzyme Mix and 0.5 μL of each target transcript primer, 0.2 μL target probe, 0.2 μL reference gene primers, 0.2 μL reference transcript probe, and 2 μL total RNA in a final volume of 10 μL. Reverse transcription was carried out at 48 °C for 10 min and then at 95 °C for 10 min. The samples were then amplified for 45 cycles at 95 °C for 10 s and then at 60 °C for 40 s. Transcript levels of the target genes were normalized to the level of the reference β-actin gene (*Actb*).

### 4.5. Analysis by Western Blotting

The glomeruli were isolated using a sieve, lysed in buffer (20 mM Tris, 140 mM NaCl, 2 mM EDTA, 10% glycerol, and 0.5% Triton X-100) containing protease inhibitor cocktail for 30 min, and centrifuged at 13,000× *g* at 4 °C for 20 min. Equal amounts of total protein were denatured (98 °C, 5 min) and then electrophoresed on a 10% sodium dodecyl sulfate-polyacrylamide gel. The proteins were transferred to PVDF membranes (2 h at 200 mA). The membranes were blocked (5% BSA, 0.05% Tween-20 in TBS) for 1 h at room temperature. The membranes were then incubated overnight at 4 °C with a primary antibody (rabbit anti-VEGF-A, 1:500; Merck KGaA, Darmstadt, Germany, Cat. No. AB1876-I; rabbit anti-β-actin, 1:1000, Sigma-Aldrich, Saint Louis, MO, USA, Cat. No. A2066). Membranes were washed (0.01% Tween-20 in TBS) and exposed to horseradish peroxidase-conjugated secondary antibody (anti-rabbit IgG; BD Pharmingen, Franklin Lakes, NJ, USA, Cat. No. 554021) for 1 h at room temperature. Super Signal West Pico PLUS chemiluminescent substrate (Thermo Fisher Scientific, Waltham, MA, USA) was used to detect reaction products. The GelDoc-It imaging system (UVP) was used to analyze and archive the membranes. The background signal was subtracted using the rolling disk method.

### 4.6. Analytical Methods

Blood glucose was measured using an Accu-Chek™ Performa glucometer (Roche, Basel, Switzerland), and urine volume was measured gravimetrically. The concentrations of rat sVCAM-1 (MyBioSource, San Diego, CA, USA, Cat. No. MBS762680), rat sICAM-1 (MyBioSource, San Diego, CA, USA, Cat. No. MBS266128), rat albumin (AssayMax, St. Charles, MO, USA, Cat. No. ERA3201-1), rat VEGF-A (Thermo Scientific, Waltham, MA, USA, Cat. No. ERVEGFA), rat thrombin–antithrombin (TAT) complex (Novus Biologicals, Easter Ave Centennial, CO, USA, Cat. No. NBP2-68132) and nucleosomes (Roche, Basel, Switzerland, Cat. No 11 774 425 001) were measured using immunoenzymatic assays. The enzymatic method was used to measure creatinine (Wiener lab., Rosario, Santa Fe, Argentina) and urea (BioSystems, Barcelona, Spain) concentrations. Total protein was measured with a biuret reagent.

### 4.7. eGFR Analysis

GFR was estimated (eGFR, µL/min) on the basis of serum creatinine < 52 µmol/L and urea concentrations according to the published formula [54]:

eGFR = 880 × W^0.695^ × C^−0.660^ × U^−0.391^ (1)

where W is weight (g), C is creatinine concentration (µmol/L) and U is urea (mmol/L).

### 4.8. Nutrient Variable Analysis

The variables were calculated using the following equations: feed efficiency ratio = (body weight gain/feed intake) × 100; food utilization efficiency for body weight = weight gain/kcal intake per day; specific rate of body mass gain = (final body weight − initial body weight)/initial body weight.

### 4.9. Statistical Analysis

Statistica 13.3 (TIBCO Software, Palo Alto, CA, USA) and GraphPad Prism 4.0 (GraphPad Software, Boston, MA, USA) were used for statistical analysis. Power analysis was used to determine the number of animals in a given group. The number of animals in a group was determined using a parameter indicative of renal injury, urinary albumin excretion. Group size was determined using Statistica 13.3 software. The Shapiro–Wilk test was used to check the normality of the distribution of variables; continuous variables are expressed as mean ± SE (standard error) or median and 25th and 75th percentiles. Unpaired *t*-test or Mann–Whitney U test were used to determine the statistical significance of differences between groups. To compare the values of two related samples, a paired *t*-test or Wilcoxon test was used. The two-way ANOVA was used to examine the possible effect of suramin on the observed differences between the groups of healthy and diabetic rats, followed by post hoc analysis using the Tukey test. Regarding non-parametric data, it was decided to compare groups using the Kruskal–Wallis test, followed by post hoc analysis using Dunn’s multiple comparisons test. Differences were considered significant at *p* < 0.05.

### 4.10. Materials

Triton X-100 and streptozotocin were purchased from Merck KGaA (Darmstadt, Germany). Suramin sodium was purchased from Santa Cruz Biotechnology (Dallas, TX, USA, Cat. No. SC-507209). Protease inhibitor cocktail, acetylcholine chloride, BSA, Tween-20, and phenylephrine hydrochloride were purchased from Sigma-Aldrich (Saint Louis, MO, USA). All of the other media were purchased from Avantor Performance Material Poland S.A. (Gliwice, Poland).

## Figures and Tables

**Table 1:** Functional variables at weeks 0, 1, and 12 in non-diabetic and diabetic rats treated with suramin. Streptozotocin (60 mg/kg, *i.p.*, STZ) or citrate buffer (CON) was injected on day −7 (week 0) and suramin (10 mg/kg, *i.p.*, SUR) or saline weekly for 12 weeks.

Parameter	Week	Experimental Groups			
		CON	SUR	STZ	STZ + SUR
**Body weight, (g)**	0	266 ±10	277 ± 10	301 ± 12	282 ± 15
	1	290 ± 9 *	302 ± 10 *	279 ± 11	278 ± 9
	12	416 ± 6 *^#^	420 ± 12 *^#^	268 ± 14 *^Δ^	297 ± 25
**Diuresis, (mL/24 h)**	0	5.8 <4.3; 9.2>	7.5 <6.6; 8.4>	5.1 <4.7; 6.5>	6.9 <6.1; 8.3>
	1	8.4 ± 1.5 *	7.1 ± 0.6	93.5 ± 11.2 *^Δ^	90.6 ± 12.2 *
	12	9.3 ± 0.6	8.5 ± 0.6 ^#^	119.5 ± 10.2 *^Δ^	130.8 ± 11.6 *^#^
**Water intake, (mL)**	0	21.0 <20.6; 25.4>	21.3 <19.0; 24.7>	21.2 <16.1; 25.9>	26.1 <22.8; 26.7>
	1	26.5 ± 1.8 *	22.8 ± 3.9	101.9 ± 9.9 *^Δ^	107.0 ± 11.5 *
	12	21.0 ± 1.3 ^#^	20.4 ± 3.5	128.2 ± 10.9 *^Δ^	142.0 ± 11.4 *^#^
**Blood glucose, (mg/dL)**	0	123 ± 3	125 ± 7	116 ± 5	123 ± 5
	1	116 ± 1 *	125 ± 2	486 ± 33 *^Δ^	484 ± 24 *
	12	115 <106; 122>	126 <109; 134>	600 <525; 600> *^Δ^	586 <551; 600> *^#^

The results are demonstrated as the mean ± standard error of the mean or median and 25th–75th percentiles, *n* = 7 per group. Statistical significance: * *p* < 0.05 vs. week 0 in the same group (paired *t*-test/Wilcoxon test), ^#^ *p* < 0.05 vs. week 1 in the same group (paired *t*-test/Wilcoxon test), ^Δ^ *p* < 0.05 vs. CON in the same week (unpaired *t*-test/Mann–Whitney U test).

**Table 2:** Nutritional variables at weeks 0 and 12 in non-diabetic and diabetic rats treated with suramin. Streptozotocin (60 mg/kg, *i.p.*, STZ) or citrate buffer (CON) was injected on day −7 (week 0) and suramin (10 mg/kg, *i.p.*, SUR) or saline was injected once a week for 12 weeks.

Parameter	Week	Experimental Groups			
		CON	SUR	STZ	STZ + SUR
**Food intake, (g)**	0	18.5 <17.0; 21.0>	20.3 <17.9; 22.5>	19.3 <18.4; 29.8>	26.3 <24.8; 28.1>
	12	16.2 ± 1.1	18.1 ± 2.0	31.9 ± 1.7 *^Δ^	34.5 ± 1.4 *
**Specific rate of body mass gain, (g/kg)**	12	443.0 ± 42.6	396.1 ± 54.2	−40.4 ± 25.7 ^&^	80.6 ± 111.1
**Feed efficiency ratio**	12	52.61 ± 5.03	45.55 ± 5.71	−3.15 ± 1.95 ^&^	4.42 ± 6.86
**Efficiency of food utilization for body weight, (g/cal)**	12	0.19 ± 0.02	0.17 ± 0.02	−0.01 ± 0.01 ^&^	0.02 ± 0.02

The results are demonstrated as the mean ± standard error of the mean or median and 25th–75th percentiles, *n* = 7 per group. Statistical significance: * p < 0.05 vs. week 0 in the same group (paired *t*-test/Wilcoxon test), ^Δ^ *p* < 0.05 vs. CON (unpaired *t*-test), ^&^ *p* < 0.0001 vs. CON (unpaired *t*-test).

**Table 3:** The primer sequences and TaqMan probes.

Gene Transcript	Accession No.	Oligonucleotide Sequence 5′-3′	Universal ProbeLibrary Probe
*rFlt1* (*Vegfr1*)	D28498	(F) cagtttccaagtggccagag	#22
		(R) aggtcgcgatgaatgcac	
*rFlk1* (*Vegfr2*)	U93306	(F) gagacccgcgttttcaga	#65
		(R) aagaacaatatagtctttgccatcc	
*Actb*	Universal ProbeLibrary Rat Actb Gene Assay (Roche, Cat #05046203001)		

## Data Availability

The data presented in this study are available on request from the corresponding author.
